# First-Row Late Transition Metals for Catalytic Alkene Hydrofunctionalisation: Recent Advances in C-N, C-O and C-P Bond Formation

**DOI:** 10.3390/molecules22111901

**Published:** 2017-11-04

**Authors:** Sophie Bezzenine-Lafollée, Richard Gil, Damien Prim, Jérôme Hannedouche

**Affiliations:** 1 Institut de Chimie Moléculaire et des Matériaux d’Orsay (ICMMO), UMR 8182, University of Paris-Sud, F-91405 Orsay, France; sophie.bezzenine@u-psud.fr (S.B.-L.); richard.gil@u-psud.fr (R.G.); 2 Institut Lavoisier de Versailles (ILV), UMR 8180, Université Versailles Saint-Quentin, F-78035 Versailles, France; damien.prim@uvsq.fr; 3 Centre National de la Recherche Scientifique (CNRS), UMR8000, Laboratoire de Chimie Physique, F-91405 Orsay, France

**Keywords:** hydrofunctionalisation, first-row late transition metals, unactivated alkenes, amines, alcohols, phosphorus compounds

## Abstract

This review provides an outline of the most noteworthy achievements in the area of C-N, C-O and C-P bond formation by hydroamination, hydroalkoxylation, hydrophosphination, hydrophosphonylation or hydrophosphinylation reaction on unactivated alkenes (including 1,2- and 1,3-dienes) promoted by first-row late transition metal catalytic systems based on manganese, iron, cobalt, nickel, copper and zinc. The relevant literature from 2009 until mid-2017 has been covered.

## 1. Introduction

The broad relevance of nitrogen-, oxygen- and phosphorus-containing compounds in numerous areas such as, for example, catalysis, bulk and fine chemicals, biological, pharmaceutical or coordination chemistry, has stimulated the attention of the scientific community to develop more effective and viable strategies for their preparations. Among them, the catalysed addition of a N-H, O-H or P-H bond onto an unactivated olefin is one of the most promising approach for the development of a powerful synthetic method with low-economic and -environmental impacts. Strictly speaking, this catalytic alkene hydrofunctionalisation approach offers a step- and atom-economical route for the syntheses of these compounds from relatively inexpensive and available starting materials [1,2,3,4,5,6,7]. Additionally, the proper catalyst design opens the door for a potential control of the chemo-, regio- and stereoselectivity of this catalytic process. However, regardless these stimulating intrinsic properties, the exclusive formation of the Markovnikov or *anti*-Markovnikov hydrofunctionalisation product by a perfect control of the reaction regiochemistry remains very demanding. As a general trend, the catalysed N-H and O-H addition reactions across unactivated carbon-carbon double bond afford the Markovnikov adduct while the corresponding P-H addition reactions proceed by an *anti*-Markovnikov regioselectivity. Only a few reports display the opposite regiochemistry for the appropriate transformation [8,9,10,11,12,13,14,15,16] highlighting the challenges to attain both synthetically valued regioisomers. Another central concern of this domain is the stereochemistry control during the carbon-heteroatom bond formation of the Markovnikov pathway. For example, the intermolecular hydroamination of simple amines and simple aliphatic olefins in a highly regio- and enantioselective fashion still remain to be unlocked [17,18] as is the enantiocontrol in alkene hydroalkoxylation [14,19]. The last few years have been the witness of an increasing interest for the development of inexpensive, first-row late transition metal-based catalysts to address some of these issues and extend the potential of the catalytic alkene hydrofunctionalisation methodology. This review will summarise the more recent and relevant advances in the area of C-N, C-O and C-P bond formation by hydroamination, hydroalkoxylation, hydrophosphination, hydrophosphonylation or hydrophosphinylation reaction on unactivated alkenes (including 1,2- and 1,3-dienes) promoted by first-row late transition metal catalytic systems based on manganese, iron, cobalt, nickel, copper and zinc. The addition reactions of water on unactivated alkenes are excluded from this review and the readers are invited to refer to published reviews on the topic [20,21]. It is also out of the scope of this review to cover all the field activity and, in this context, literature coverage has been restricted from 2009 until mid-2017, although a few illustrative earlier reports are mentioned. The achievements in the field are described below according to the nature of the metal and the type of alkene hydrofunctionalisation reaction.

## 2. Manganese

### 2.1. Hydroamination and Hydroalkoxylation

To the best of our knowledge, despite the nascent development of manganese in catalysis [22] and the involvement of this metal in related catalytic alkene hydrofunctionalisation processes such as hydrohydrazination or hydratation [23], no report of manganese-catalysed hydroamination or hydroalkoxylation of unactivated alkenes has been published yet.

### 2.2. Hydrophosphination

**Scheme 1:**
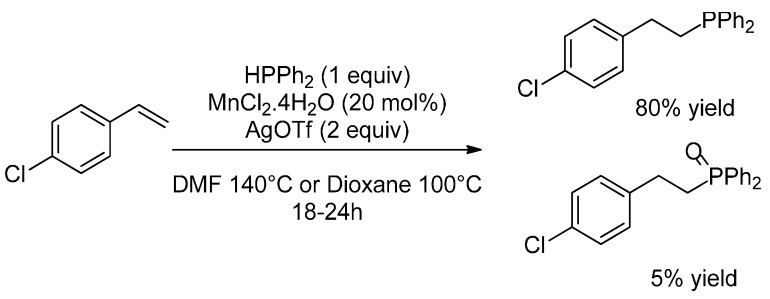
*Anti*-Markovnikov manganese-mediated hydrophosphination of 4-chlorostyrene.

The use of manganese salts in the context of alkene hydrophosphination is only scarcely described. Indeed, Corma and co-workers used MnCl_2_·4H_2_O in combination with AgOTf to promote the hydrophosphination reaction of 4-chlorostyrene and diphenylphosphine as the sole example (Scheme 1) [24]. Although the overall yield is good, the authors reported the formation of a mixture including the expected *anti*-Markovnikov product in 80% yield and the corresponding phosphine oxide in 5% yield.

## 3. Iron

### 3.1. (Formal) Hydroamination

**Scheme 2:**
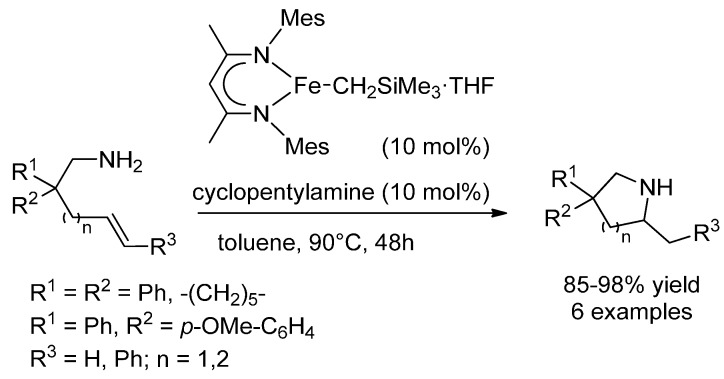
Iron-catalysed cyclohydroamination of primary amines tethered to unactivated alkenes.

Preliminary investigations in the field have brought into light the benefit of iron(III) salts as Lewis acid catalysts for the C-N bond formation by the addition of electronically deficient amines, mainly as *N*-tosylamines, onto unactivated alkenes via a hydroamidation process [25,26,27,28,29]. However, these early studies are limited to electronically biased amines and problems of regioselectivity have been noticed in some cases [25]. The first disclosure of iron-promoted hydroamination reaction of electronically unbiased amines appeared in early 2014 [30]. In this work by the Hannedouche group, it was demonstrated that structurally defined β-diketiminatoiron(II)-alkyl complexes were particularly efficient as well-defined low-coordinate catalysts for the selective intramolecular hydroamination of primary amines tethered to unactivated alkenes, in the presence of cyclopentylamine as co-catalyst (Scheme 2). Beside this iron-based methodology provides the corresponding cyclised products in good to excellent yields, it does not proceed with aminoalkenes without a Thorpe-Ingold effect or with 1,2-dialkyl-substitution on the pendant alkene. Mechanistic studies support a stepwise σ-insertive mechanism including a migratory 1,2-insertion of the alkene into an iron-amido bond of an isolable amido iron complex, and an aminolysis step of the resulting insertive product as the rate-limiting step. The presence of a catalytic amount of cyclopentylamine as a poorly noncyclisable primary amine reduces the formation of side products resulting from a β-H elimination pathway and consequently allows the observation of a high reaction selectivity for the hydroamination over an oxidative amination pathway.

**Scheme 3:**
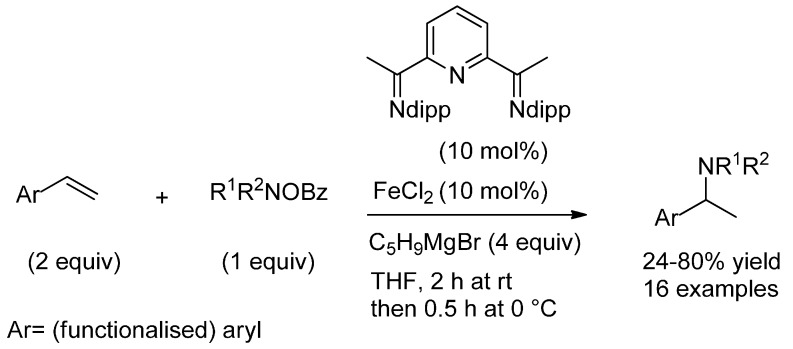
Markovnikov regioselective iron-catalysed electrophilic amination of functionalised vinylarenes.

In the same year, the first iron example of C-N bond formation on unactivated alkenes by an umpolung electrophilic amination as an alternative strategy to the classical hydroamination was reported by Yang and co-workers [31]. Experimental investigations reveal that a catalytic combination of iron(II) dichloride and a 2,6-diiminopyridine ligand in the presence of four equivalents of cyclopentylmagnesium bromide as reducing agent was efficient for the regioselective formal hydroamination of functionalised vinylarenes with hydroxylamine esters as the electrophilic nitrogen source (Scheme 3). This procedure is effective for the formal hydroamination of a range of electron-poor and -rich substituted vinylarenes and affords the corresponding branched amines in moderate to excellent yields. However, it is inappropriate for the transformation of α- and β-methylstyrenes, styrenes substituted by a trifluoromethyl or a chloro group on the phenyl ring as well as aliphatic terminal alkenes. Similarly to the more-studied copper-hydride formal hydroamination methodology (*vide infra*), a regioselective hydrometalation of the vinylarene derivative by an in situ-generated iron-hydride species may be proposed as the main elementary step of the catalytic cycle.

**Scheme 4:**
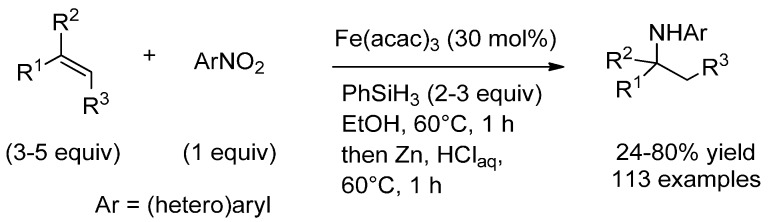
Iron-hydride promoted formal hydroamination of (poly)substituted alkenes and nitro(hetero)arenes.

In 2015, inspired by their previous works on iron hydride promoted H-atom transfer processes for radical mediated carbon-carbon bond formation from alkenes [32,33] the Baran group disclosed a gentle and useful iron-based methodology for the synthesis of (hetero)aryl secondary amines substituted by a secondary or tertiary alkyl group from mono- and polysubstituted olefins and nitro(hetero)arenes (Scheme 4) [34]. Under the optimised reaction conditions displayed in Scheme 4, a remarkable variety of hindered (hetero)aryl secondary amines were prepared under exclusive Markovnikov selectivity with low to good isolated yields by the use of iron(III) acetylacetonate (30 mol %) and phenylsilane (2–3 equiv.). The functional group tolerance of this air- and moisture compatible protocol is impressively broad (function such as nitriles, amides, thioethers, amines, ketones, alcohols, halides, boronic acids or triflates are compatible) and this is a great advantage for further derivatisations in an orthogonal manner to the classical C-N bond formation methodologies. However, this protocol, which needs the use of an excess of alkene, is limited to nitro (hetero)arenes and is not applicable to α-substituted styrenes, 2-nitropyridines, nitroimidazole, and nitrophenyl having orthoesters or free thiols or alcohols. In this process, iron-hydride species (generated in situ from iron(III) acetylacetonate and phenysilane) mediate the formal single-electron reduction of both partners, the nitro(hetero)arene and the olefin into, respectively, a nitroso(hetero)arene and an alkyl radical. Subsequent radical attack of one equivalent of the alkyl radical (generated from the olefin) on the nitroso(hetero)arene furnishes a O-centred radical species which is further reduced to provide the formal hydroamination product [23,35]. One year later, the groups of Shenvi [36] and Thomas and Shaver [37,38] brought some modifications to the initial conditions developed by Baran and co-workers by using isopropoxy(phenyl)silane (instead of phenylsilane) and an amine–bis(phenolate)iron(III) complex (instead of iron(III) acetylacetonate), respectively. Under these modified conditions, the catalyst loading was decreased and the reaction temperature was reduced to room temperature.

### 3.2. Hydroalkoxylation

**Scheme 5:**
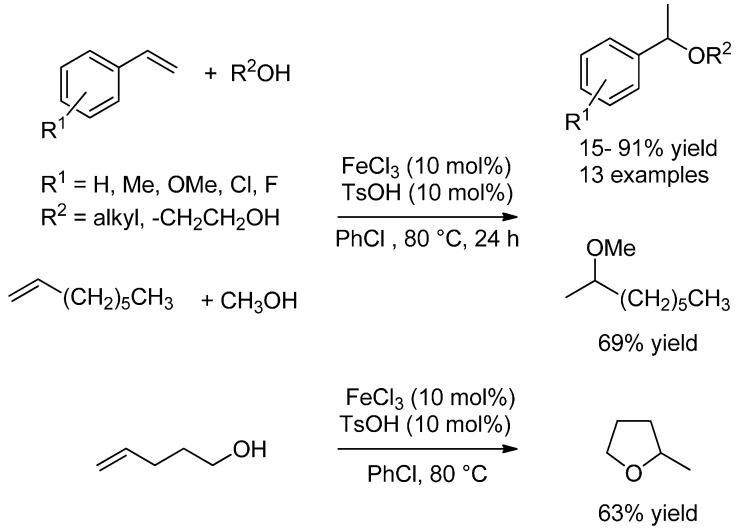
Inter- and intramolecular hydroalkoxylation of alkenes catalysed by iron chloride in the presence of TsOH.

The use of iron salts as Lewis acids in inter- and intramolecular hydroalkoxylation of alkenes has emerged over the past decade [39]. Efficient, inexpensive and non-toxic iron(III) chloride catalyst can be employed in the presence of different additives like silver triflate or *p*-toluenesulphonic acid and these catalytic systems are tolerant with various functional groups. In 2007, the Takaki group was the first to evidence the catalytic activity of cationic iron complexes for the intramolecular hydroalkoxylation of various alkenes with the catalytic system FeCl_3_/AgOTf [40]. A few years later, the intermolecular version of this transformation with aliphatic alcohols and styrene derivatives and linear alkenes was reported by Zhou et al., using the combination FeCl_3_/TsOH (Scheme 5). This catalytic system which displays Markovnikov selectivity is also effective for the intramolecular version of the reaction [41].

**Scheme 6:**
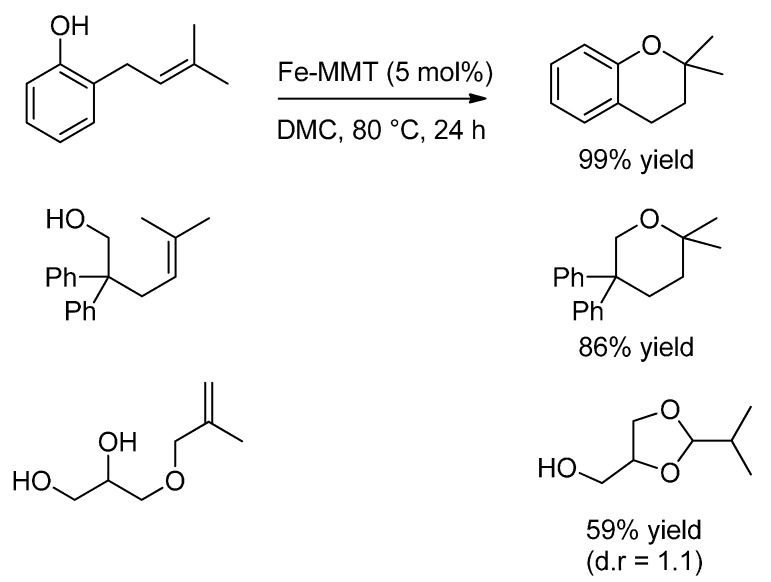
Intramolecular hydroalkoxylation of alkenes catalysed by Fe-MMT.

An interesting and recent material prepared from iron(III) chloride and Montmorillonite has been reported by Antioniotti et al. [42]. The supported catalyst Fe-MMT is very efficient in dimethyl carbonate (DMC) at 80 °C (Scheme 6) and is superior in activity and selectivity to its homogeneous equivalent. This heterogeneous catalyst can be reused several times without loss of activity.

**Scheme 7:**
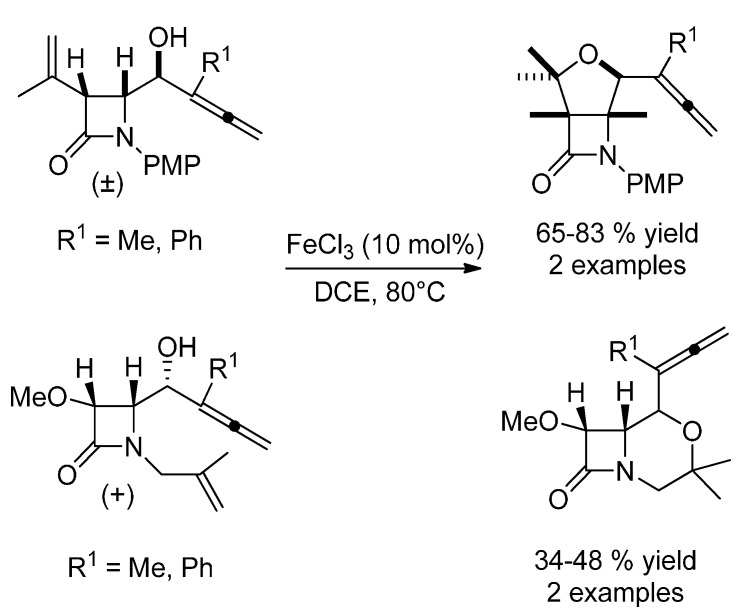
FeCl_3_ catalysed hydroalkoxylation of β-lactam enallenols.

In 2011, during the oxycyclisation study of β-lactam enallenols, Alcaide and Almendros have illustrated an efficient chemodivergent metal-controlled methodology [43]. The cyclisation of enallenols catalysed by FeCl_3_ is in favour of the alkenol activation whereas the chemoselectivity with precious metal salts—[PtCl_2_ (CH_2_ = CH_2_)]_2_ and AuCl_3_—affords the allene cycloisomerisation adduct. For the first time, a total selectivity in favour of an olefin moiety vs. allene functionality has been described. Moreover with FeCl_3_, the exclusive formation of the β-lactam-tetrahydrofuran hybrid was observed from a regiospecificity for the five-membered rings (Scheme 7). With BiCl_3_, InCl_3_ or HfCl_4_, stoichiometric amounts of activators are needed to reach complete conversions. It is worth noting that in the case of β-lactam enallenols presenting tetrasubstituted olefins, carbonyl group becomes more electrophilic than the tetrasubstituted alkene leading to lactones [44].

**Scheme 8:**
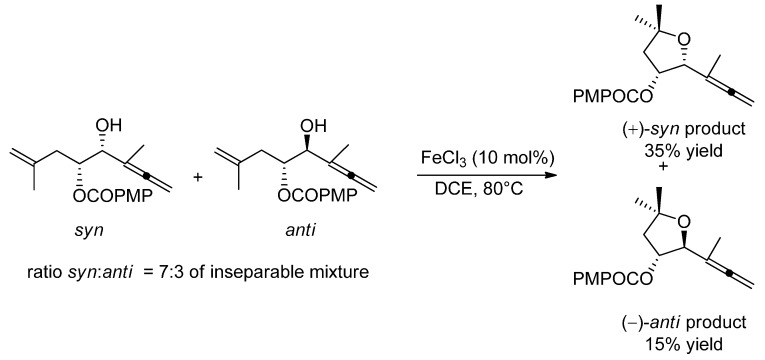
FeCl_3_ catalysed hydroalkoxylation of non β-lactam enallenols.

During the application of this chemodivergent metal-controlled methodology to non-β-lactam enallenols, full chirality transfer from *syn* and *anti* enallenols was noticed (Scheme 8) [43]. However, a sequence involving a carbocyclisation and a dehydration reaction was observed with an enallenol moiety linked to an aromatic ring.

**Scheme 9:**
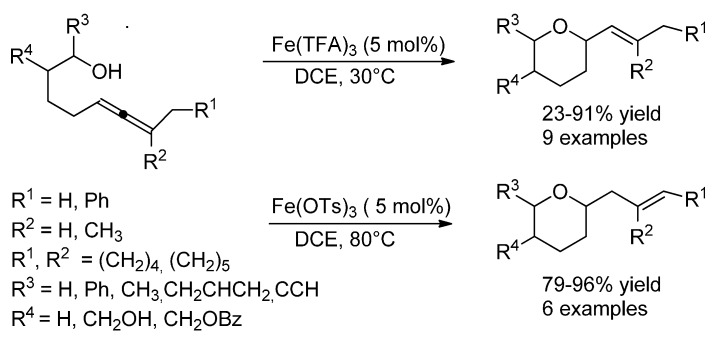
Hydroalkoxylation reaction catalysed by Fe(TFA)_3_ and Fe(OTs)_3_ for the synthesis of tetrahydropyrans.

In 2012, Kang et al., disclosed a highly efficient catalytic system for the hydrofunctionalisation of trialkylated δ-allenyl alcohols and amines [29]. According to the nature of the iron counterion and the reaction conditions, an isomerisation of the hydroalkoxylation product may or not be observed as represented in Scheme 9. As mentioned in Section 3.1, this catalytic system was also efficient for the cyclohydroamination of *N*-tosyl aminoallenes [29].

**Scheme 10:**
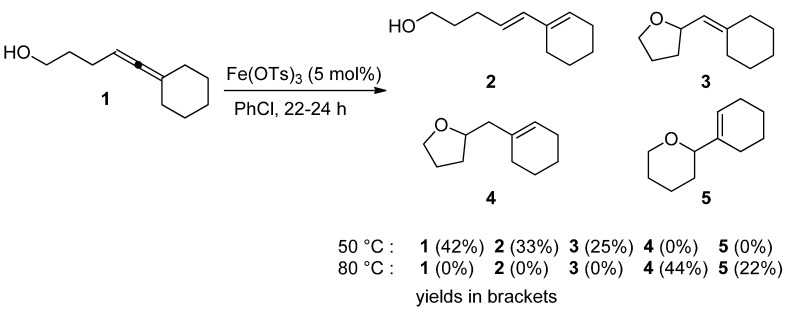
*Endo*/*exo*-methylene selectivity of intramolecular hydroalkoxylation reaction of allenols catalysed by Fe(OTs)_3_.

More recently Kang et al., have shown that the temperature influences the isomerisation and the *endo*/*exo*-methylene selectivity of hydroalkoxylation products of cyclohexyl-substituted allene catalysed by iron(III) tosylate (Scheme 10) [45]. At 50 °C, a partial isomerisation of allenol **1** in **2** occurs and during the catalytic process, **1** is converted into tetrahydrofuran **3** via a 5-*exo*-trig cyclisation. At 80 °C, the presence of the catalyst induces an isomerisation in favour of **4**. The dienyl alcohol **2** can be used at 80 °C as a starting material leading to **4** and **5** in respectively 38% and 32% yield. The higher ratio of **5** in the reaction with **2** could be explained by an alkene-iron activation passing by a 6-*endo*-trig cyclisation in favour of the tetrahydropyran derivative.

**Scheme 11:**
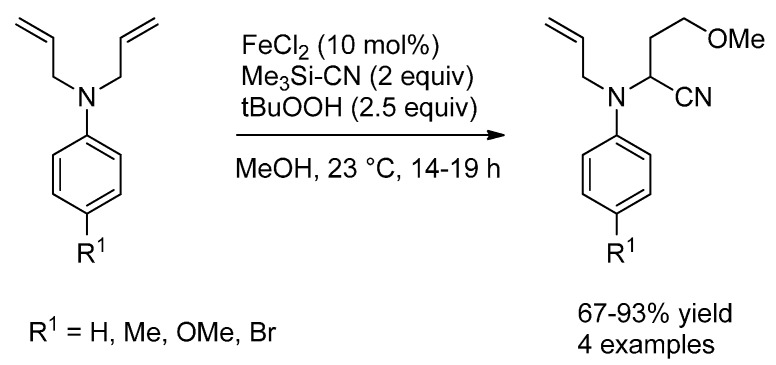
Sequential oxidative cyanation/hydroalkoxylation of tertiary *N*,*N*-diallylamines catalysed by FeCl_2_.

Because of the stability of cationic intermediates, a total Markovninov selectivity is mainly reported in the literature. In order to reverse this selectivity, stabilised radical species must be formed to allow this selectivity change. In 2015, Ofial et al., have described an original sequential oxidative α-cyanation/*anti*-Markovnikov hydroalkoxylation of tertiary allylamines catalysed by iron(II) chloride (Scheme 11) [46]. The catalyst is involved in the first step of the transformation in which cyanide ion attacks the iminium functionality derived from the oxidation of the starting amine. The α-cyano-amine undergoes a second step of oxidation leading to a radical species stabilised by a captodative effect. Finally, methanol can react on the allylic radical species in a Michael-type approach giving a formally *anti*-Markovnikov intermolecular hydroalkoxylation product.

### 3.3. Hydrophosphination

**Scheme 12:**
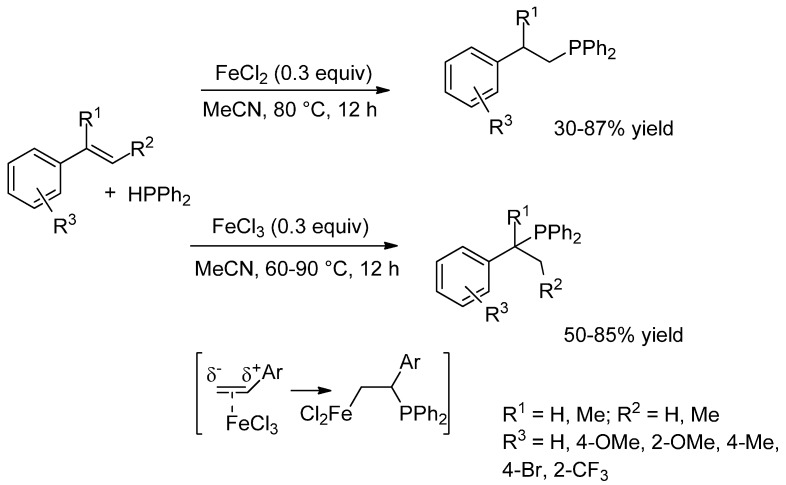
FeCl_2_-versus FeCl_3_-catalysed hydrophosphination of styrene derivatives: a switchable regioselectivity.

Recently, Gaumont, Taillefer et al., have reported the appealing hydrophosphination of alkenes using simple iron salts [16]. Indeed, FeCl_2_ and FeCl_3_ can promote the hydrophosphination without the presence of any ligand. Interestingly, the selective α- or β-phosphinylation of styrenes can be obtained depending on the metal oxidation state. Moving from Fe(II) to Fe(III) allowed switching the selectivity. If the former direct the formation of *anti*-Markonikov adduct, Fe(III) mediates the formation of a benzylic stereogenic centre (Scheme 12). Electronic effects as well as strict anhydrous conditions only affect the hydrophosphination issues with a lesser extent. The mechanism and switch of selectivity observed remain unclear. As stated by the authors, the difference of Lewis acidity between Fe salts might be responsible for the selectivity observed. Further, the polarised π-complex in Fe(III) promoted reactions may originate from intramolecular stabilisation.

**Figure 1:**
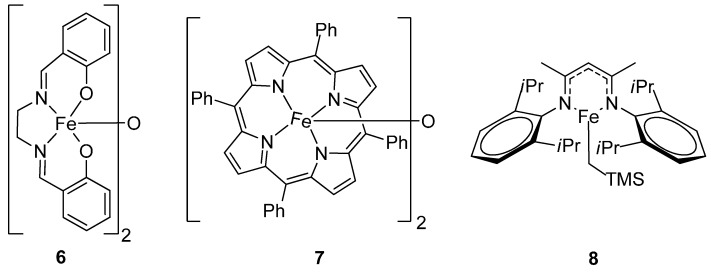
β-diketiminate-, salen- or porphyrin-based iron complexes.

Recently Webster et al., have reported the hydrophosphination of styrene derivatives using iron-based catalysts at very low catalytic loadings (0.5 mol %) [47,48,49]. As shown in Figure 1, β-diketiminate-, salen- or porphyrin-based iron complexes have been successfully applied towards alkene hydrophosphination.

**Scheme 13:**
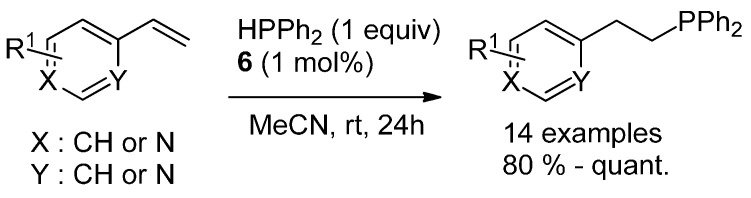
*Anti*-Markovnikov hydrophosphination using a salen-based iron complex.

Air stable salen-type iron complex **6** was efficient in the hydrophosphination of various styrenes and vinylpyridines (Scheme 13) [47]. Indeed, hydrophosphination proceeds with low catalyst loading (0.5%) at room temperature to afford the *anti*-Markovnikov adduct. In contrast, less active alkenes such as allylbenzene and alkenes yielded only traces amounts of product. Likewise styrene poorly reacts with hindered phosphines such as HPCy_3_ or monophosphines such as H_2_PPh.

**Scheme 14:**
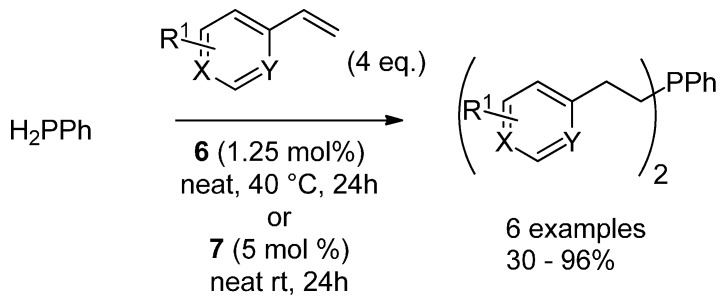
Double hydrophosphination of styrenes derivatives promoted by iron complexes.

**Scheme 15:**
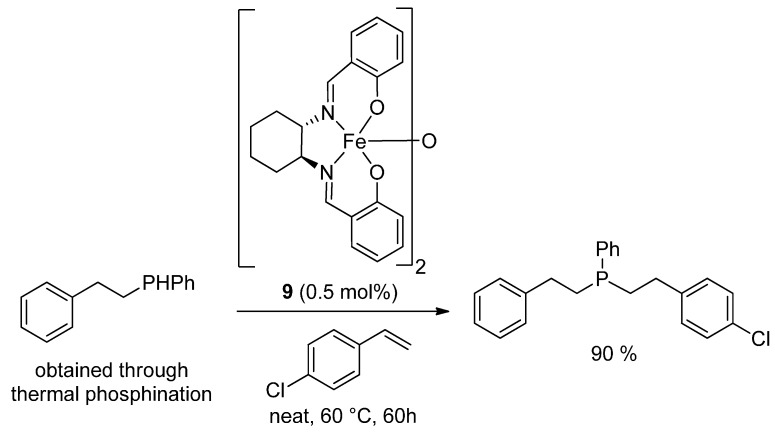
Sequence towards unsymmetrical hydrophosphination of styrene derivatives.

Precatalysts **6** and **7** allowed a double alkene hydrophosphination reaction [48,49]. This transformation has been successfully applied to variously substituted styrenes or vinylpyridines and H_2_PPh. Interestingly, a two-step sequence towards unsymetrical phosphanes was developed using a thermal followed by a metal-catalysed hydrophosphination (Scheme 14). The first step proceeded with high selectivity and functional group tolerance. The use of 0.5 mol % of µ-oxo iron complex **9** derived from Jacobsen-type ligand led to unsymetrical phosphanes in 90% yield (Scheme 15). Unfortunately no stereoinduction was obtained.

**Scheme 16:**
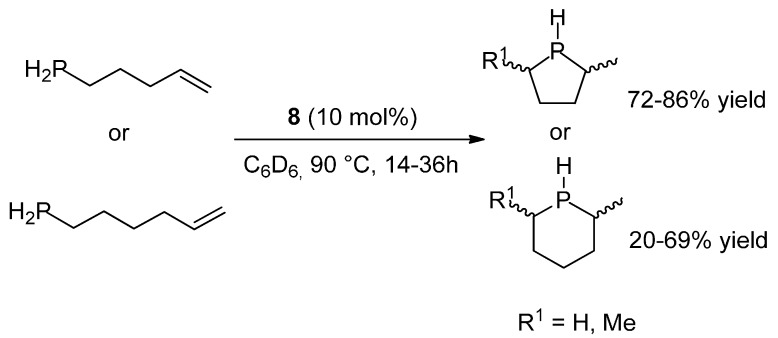
Intramolecular hydrophosphination of alkenes promoted by a β-diketiminatoiron(II) complex **8**.

The same research group reported the use of β-diketiminate iron(II) complexes for the intramolecular hydrophosphination of alkenyl phosphines (Scheme 16) [49,50]. Non-activated phosphino-alkenes readily undergo cyclisation to afford phospholes and phosphinanes as mixtures of isomers in fair to high yields.

## 4. Cobalt

### 4.1. Formal Hydroamidation

**Scheme 17:**
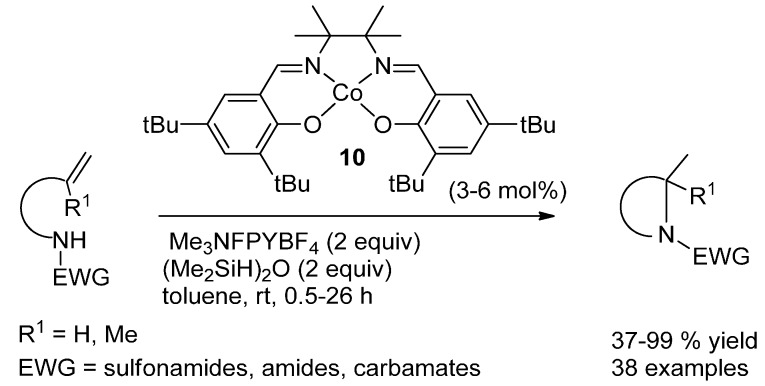
Cobalt-promoted alkene cyclohydroamidation of *N*-protected amines.

From their brilliant achievement on cobalt-catalysed intermolecular hydroalkoxylation of unactivated alkenes [51] (*vide infra*), the Shigehisa group has demonstrated that salen-stabilised cobalt(II) complex **10** in the presence of an excess of a fluorine and hydride source, respectively, *N*-fluoro-2,4,6-trimethylpyridinium tetrafluoroborate (Me_3_NFPYBF_4_) and 1,1,3,3-tetramethyldisiloxane ((Me_2_SiH)_2_O), was particular effective as catalyst for the intermolecular formal hydroamidation of a large variety of functionalised *N*-protected aminoalkenes at room temperature (Scheme 17) [52]. The substrate scope and the functional group tolerance of this methodology is remarkably wide affording three-, five-, six-, and even seven-membered ring compounds from aminoalkenes having various electron-withdrawing groups on the nitrogen atom. Regrettably, this system is inefficient for the cyclisation of free or *N*-benzyl amines and internal alkenes and progress is still needed in this direction to offer a “truly” hydroamination protocol. From the initial mechanistic study conducted on the alkene hydroalkoxylation catalysed by a similar cobalt system [51] as well as some related literature [23], it might be postulated that the reaction proceeds by a regioselective hydrometallation of the alkene by a Co(III)-H species, followed by homolytic cleavage of the resulting Co(III)-alkyl species. Next, oxidation of the ensuing carbon radical intermediate would lead to the formation of a carbocation intermediate, which subsequently endures a nucleophilic attack by the *N*-protected amine.

### 4.2. Hydroalkoxylation

**Scheme 18:**
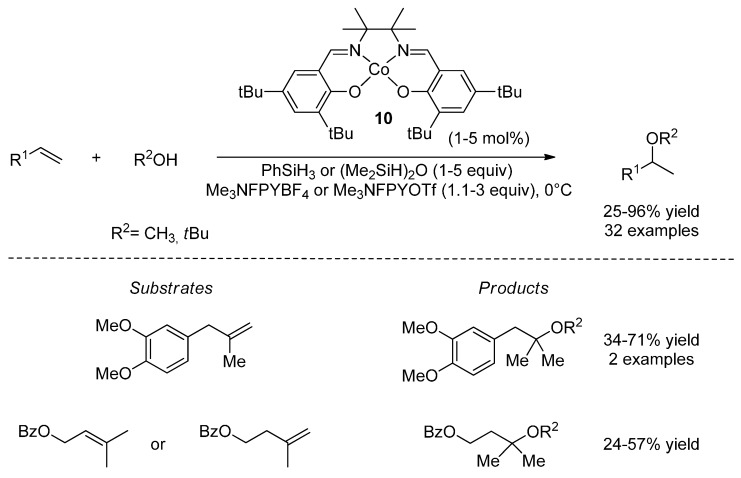
Cobalt-promoted intermolecular hydroalkoxylation of alkenes.

The remarkable catalytic system composed of the cobalt complex **10**, a silane and a *N*-fluoropyridinium salt displayed in Scheme 16 for the alkene cyclohydroamination was initially employed for intermolecular hydroalkoxylation of unactivated alkenes (Scheme 18) [51]. After the optimisation of the salen type cobalt complex and the fluoride source, Shigehisa and Hiroya et al., have shown that this catalytic system was very efficient for the intermolecular hydroalkoxylation implying various functionalised terminal olefins (that present TBS, PMB, acetal, esters, amides, bromo, nitro, tosylates, heterocycles, sulphur atoms and amino surrogates) but also for di- and tri-substituted olefins.

**Scheme 19:**
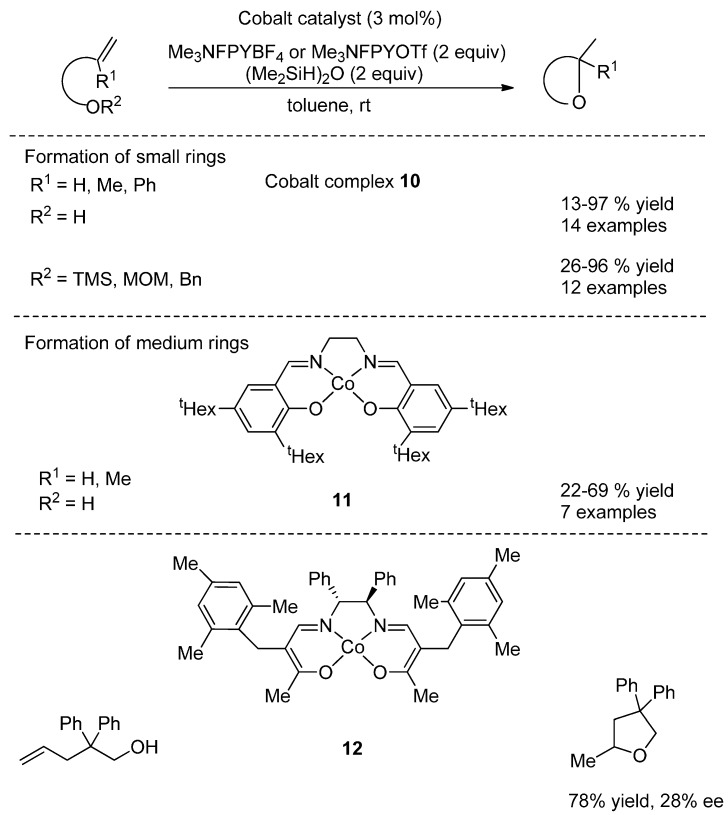
Cobalt-promoted intramolecular hydroalkoxylation of alkenyl alcohols.

In 2016, the same research group extended the application of this catalytic system to intramolecular hydroalkoxylation of alkenols (Scheme 19) [53]. Various cyclic ether containing five- or six-membered ring were obtained in good to excellent yields and good functional groups tolerance. After the optimisation of the ligand structure of cobalt complex **11**, this method was also applied to the formation of seven-, eight- and nine-membered ring ethers from alkenyl mono- and di-substituted olefin. In the case of small ring compounds (five- and six-membered rings), the authors have shown that it was possible to carry out the deprotective cyclisation of some protected alkenyl alcohols to directly afford the corresponding cyclic ethers. The involved protecting groups are TBS (tert-butyldimethylsilyl), MOM (methoxy acetal), MEM (methoxyethoxymethyl acetal), BOM (benzyloxy acetal) Bn (benzyl) and Me (methyl). This very efficient cobalt-based catalytic system for intramolecular hydroalkoxylation under mild reaction conditions was also applied for the corresponding intramolecular hydroacyloxylation reaction. It may also be noted that during their study, the authors performed the first asymmetric reaction from 2,2-diphenylpent-4-enol using the chiral cobalt complex **12** (Scheme 19). The corresponding hydroalkoxylation product was obtained with 28% of non-optimised enantiomeric excess. These studies allow the authors to propose a reaction mechanism closely related to the one mentioned for the cobalt-catalysed hydroamination of alkenes (Section 4.1). After a regioselective alkene hydrometallation by a Co(III)-H species, followed by homolytic cleavage of the ensuing Co(III)-alkyl species, a single-electron oxidation of the carbon radical and intramolecular nucleophilic trapping by the oxygen atom might both subsequently proceed in a concerted manner [53].

**Scheme 20:**
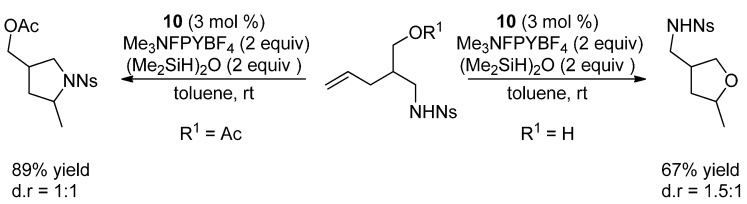
Hydroalkoxylation versus hydroamination promoted by a cobalt complex.

It is interesting to note that the selectivity favours hydroalkoxylation versus hydroamination with this catalytic system, as represented in Scheme 20 [52], although an interesting switch of selectivity can be achieved using an acetyl protecting group on the alcohol.

**Scheme 21:**
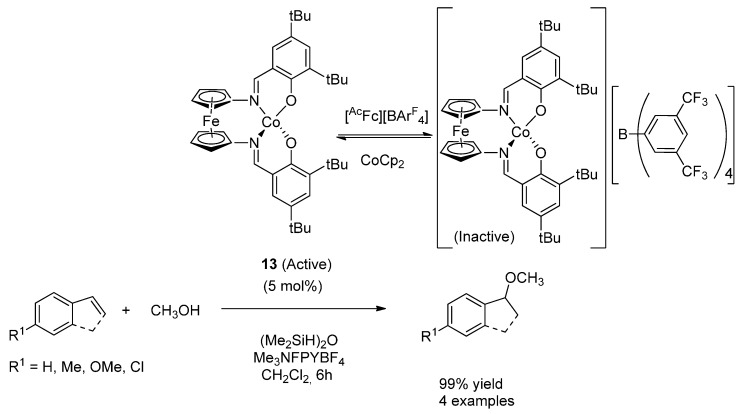
Redox switchable hydroalkoxylation of a cobalt complex.

In 2016 Diaconescu et al., described the synthesis of a novel cobalt salfen complex **13** containing a ferrocene moiety into the backbone (Scheme 21) [54]. The authors have demonstrated that this original complex presents unusual steric, electronic and electrochemical properties. This first crystallographically characterised tetrahedral cobalt salen complex undergoes two oxidation events at low potentials assigned as ligand centered. Under the conditions of Shigehisa and Hiroya et al., and in the presence of a silane and an electrophilic fluorinating agent in trifluorotoluene, this complex was able to catalyse some hydroalkoxylation reactions of unactivated olefins but for a smaller scope (only for styrenes) compared to other salen cobalt complexes. Concerning the mechanism, the authors suggest that complex **13** can adopt a square pyramidal geometry in the presence of alcohol allowing it to access the Co(III) oxidation state. Unprecedented, the hydroalkoxylation reactivity could be turned off in the presence of the one-electron-oxidised species generated in the presence of acetylferrocenium tetraarylborate [^Ac^Fc][BAr^F^_4_] (ArF = 3,5-(CF_3_)_2_-C_6_H_3_). On the other hand, we can note that when the hydroalkoxylations of styrenes were performed in the presence of the corresponding zinc complex, which was synthesised in order to study the redox behaviour of Co (Salfen), no catalytic activity was observed.

## 5. Nickel

### 5.1. Hydroamination

Despite early studies in the field [55,56], the first and sole report to date of a general nickel-based methodology for the hydroamination of unactivated alkenes was published in 2002 by the Hartwig group [57]. In the course of that work, it was identified that bis(1,5-cyclooctadiene)nickel(0) in conjunction with a 1,1′-bis(diphenylphosphino)ferrocene ligand and in the presence of an acid was an efficient system for the regioselective hydroamination of 1,3-dienes and primary and secondary amines. To the best of our knowledge, no succeeding report of nickel-promoted hydroamination of unactivated alkenes has been disclosed.

### 5.2. Hydroalkoxylation

**Scheme 22:**
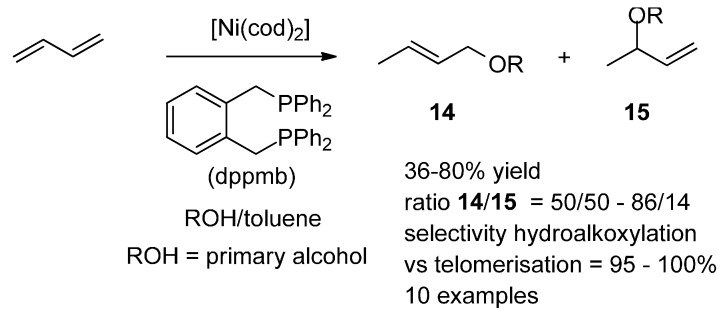
Intermolecular hydroalkoxylation of butadiene with primary alcohols catalysed by nickel.

The first example implying nickel catalyst was reported by Suisse and Sauthier et al., in 2013 [58]. They have shown that hydroalkoxylation of the 1,3-butadiene with methanol, ethanol or benzylic alcohol led to alkylbutenyl ethers with high selectivity. The catalytic intermolecular reactions were performed in the presence of [Ni(acac)_2_] and 1,4-bis(diphenylphosphino)butane (dppb) with sodium borohydride as reducing agent to generate in situ Ni(0) species. In 2016, the same group performed an interesting optimisation of this catalytic system (Scheme 22) [59]. High selectivities could be reached by replacing 1,4-bis(diphenylphosphino)butane (dppb) with 1,2-bis(diphenylphosphinomethyl)benzene (dppmb). An extension of the scope of substituted dienes was performed but a decrease of reactivity was observed. However, this study proved the efficiency of the catalytic system under mild conditions for a large range of primary alcohols including bio-sourced alcohols. In 2017, this team applied their methodology to the hydroalkoxylation of butadiene with glycerol to obtain glycerylbutenylethers (GBE) [60]. After an optimisation of the catalytic system and reaction conditions, a mixture of mono and dibutenyl glyceryl ethers was obtained in a continuous process with butadiene at atmospheric pressure. This process involves renewable substrates and the reaction was scaled up to produce multigrams of each monoethers and diethers.

### 5.3. Hydrophosphinylation

**Scheme 23:**
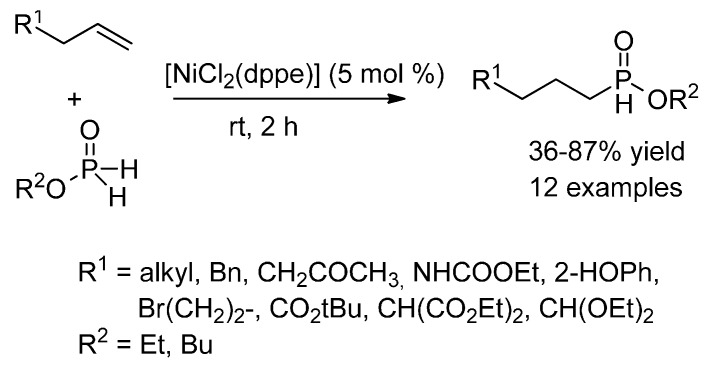
Nickel-catalysed hydrophosphinylation of unactivated linear alkenes.

Hydrophosphinylation of alkenes can alternatively be achieved using [1,2-bis(diphenyl-phosphino)ethane]dichloronickel] as the catalytic system [61]. Montchamp et al., have provided a new entry towards the formation of C-P bonds starting from terminal, internal and functionalised alkenes (Scheme 23). Interestingly, substrates such as 1,5-hexadiene selectively undergo selective mono- or bisphosphinylation. Further, the methodology also allows the easy preparation of H-phosphinates fragments. Unfortunately, combination of Josiphos or Chiraphos ligands with NiCl_2_ as the chiral catalytic system led to poor enantioselectivities (17–18% ee) by desymmetrisation.

## 6. Copper

### 6.1. (Formal) Hydroamination

**Figure 2:**
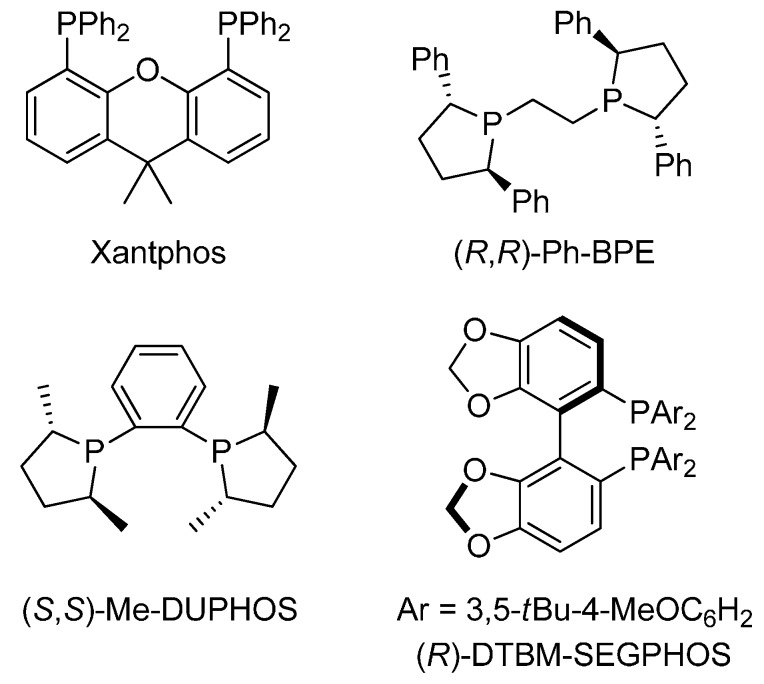
Bidentate diphosphine ligands used in the copper-catalysed hydroamination reactions.

**Scheme 24:**
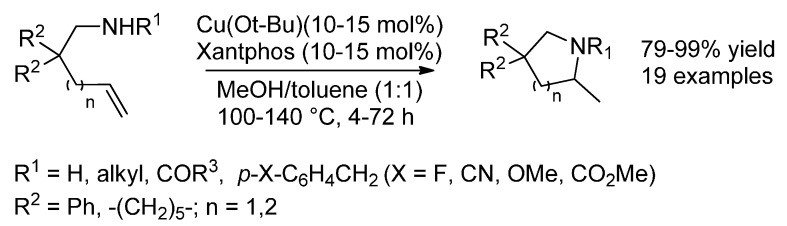
Copper (I)-catalysed cyclohydroamination of alkenes.

Although both earlier and more recent works have emphasised the usefulness of copper in the alkene hydroamination of sulphonamides [62,63,64], the first and only disclosure of an alkene hydroamination reaction of protecting-group free primary amines promoted by a copper-derived catalyst was reported in 2009 by the Sawamura group [65,66]. In this study, it was revealed that the association of copper (I) isopropoxide and the large bite angle 4,5-bis(diphenylphosphino)-9,9-dimethylxanthene (Xantphos, Figure 2) was a particularly efficient catalytic system in an alcoholic solvent for the cyclisation of primary and secondary amines (also amides) tethered to terminal alkenes affording the corresponding five- and six-membered ring nitrogen heterocycles (Scheme 24). This protocol has a relatively high functional group tolerance and chemoselectivity as no side-product from oxidative amination was observed. Mechanistically speaking, it might be proposed that the amine functionality of the aminoalkene is activated by a basic alkoxycopper species to generate a copper (I)-amido intermediate, which is subjected to a regioselective migratory alkene insertion. Subsequent alcoholysis of the resulting insertive complex would liberate the hydroamination product and regenerate the active catalyst.

**Scheme 25:**
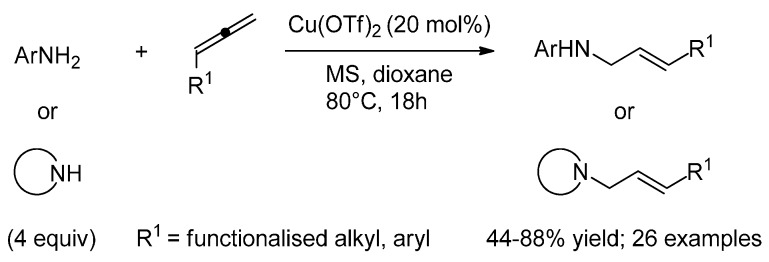
Regioselective copper(II)-catalysed intermolecular hydroamination of terminal allenes.

In 2016, the first report of copper-catalysed hydroamination of terminal allenes and cyclic secondary amines or anilines was published by Monnier and Taillefer and co-workers [67]. This methodology, which uses copper(II) triflate as catalyst and requires an excess of the nitrogen partner, is highly regioselective delivering solely the corresponding linear (*E*)-allylamines with moderate to excellent yield (Scheme 25). Moreover, halogens, free tertiary alcohols, imides and ethers are compatible functional groups on both allenes and amines.

**Scheme 26:**
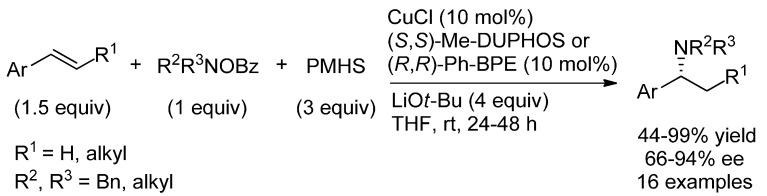
Regio- and enantioselective copper (I) hydride catalysed electrophilic amination of styrene derivatives.

In 2013, the research groups of Hirano, Miura [68] and Buchwald [69] have independently published an innovative copper-based strategy for the highly enantioselective intermolecular C-N bond formation from styrenes and hydroxylamine esters as partners, with exclusive Markovnikov selectivity. Encouraged by previous seminal studies [70], both groups brilliantly combine the well-known copper-hydride reduction chemistry [71,72] with electrophilic amination reagents [73] to put forward an efficient umpolung amination strategy, now-called formal hydroamination, as an alternative to the classical hydroamination approach. Screening experiments from the Hirano, and Miura group led to the discovery that copper(I) chloride (10 mol %) in conjunction with a chiral diphosphine ligand, (*R*,*R*)-Ph-BPE or (*S*,*S*)-Me-DUPHOS (Figure 2) (10 mol %) and in the presence of polymethylhydrosiloxane (3 equiv) and LiOt-Bu (4 equiv.) was a very efficient combination for the enantioselective Markovnikov hydroamination of functionalised styrenes and *trans*-β-substituted styrenes with *O*-benzoylhydroxylamines at room temperature, providing the addition products in good to excellent yields and with up to 94% enantiomeric excess (Scheme 26) [68]. This combination is also efficient for the conversion of strained aza- and oxa-bicyclic alkenes into the corresponding amines [74] but is unsuitable for terminal alkenes and *cis*-β-substituted styrenes.

**Scheme 27:**
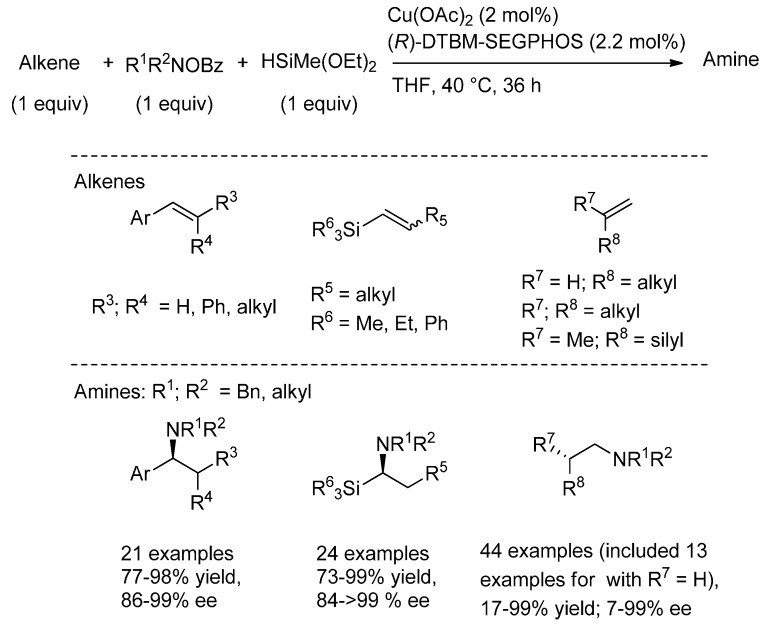
Regio- and enantioselective copper (I) hydride catalysed electrophilic amination of styrene derivatives, vinylsilanes and terminal aliphatic alkenes.

Concomitantly, the Buchwald group has published a related copper system, using copper(II) acetate (2 mol %) and chiral diphosphine ligand (*R*)-DTBM-SEGPHOS (Figure 2) (2.2 mol %) in the presence of diethoxymethylsilane (1 equiv) as hydride source, for the Markovnikov hydroamination of arylalkenes and hydroxylamine esters [69]. This catalytic system affords a broader substrate scope than Hirano and Miura’s system with a similar level of enantioselectivity. Indeed, under the reaction conditions displayed in Scheme 27, this formal hydroamination protocol is applicable to functionalised styrenes, *cis*- and *trans*-β-substituted and β,β-disubstituted styrenes affording the branched amines with high enantiomeric excesses. Additionally, linear amines can be prepared in high yields from terminal aliphatic alkenes via an exclusive *anti*-Markovnikov amination using the racemic version of this copper system [69]. This DTBM-SEGPHOS-based copper-hydride protocol has been broadened to the synthesis of structurally diverse chiral α-aminosilanes and β-chiral amines as key building blocks, from respectively, vinylsilanes [75] and 1,1-disubstituted olefins [76], with exceptional high enantioinduction. From detailed mechanistic investigations [77,78], it is proposed that the reaction proceed by an irreversible, regioselective and enantio-determining migratory olefin insertion into an in situ generated and monomeric copper(I)-hydride species. Subsequent electrophilic amination of the ensuing insertive copper complex by the amination reagent in a stereoretentive manner results in the C-N bond formation and the generation of a copper (I) carboxylate intermediate as the resting state of the catalyst. The catalytic cycle is completed by a σ-bond metathesis between the generated copper carboxylate intermediate and the silane as the rate-determining step. Electronic stabilisation of the alkylcopper intermediate by the adjacent aryl or vinyl substituent may explain the Markovnikov selectivity. In contrast, the linear amine may arise from the formation of a less crowded alkyl intermediate during the migratory insertion step.

**Scheme 28:**
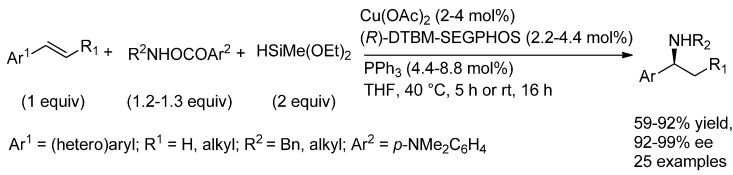
Regio- and enantioselective copper (I) hydride catalysed electrophilic amination of styrene derivatives for the preparation of chiral secondary amines.

In 2015, the use of a monoalkylating amination reagent having a 4-(dimethylamino)benzoate group instead of the previously used benzoate group allowed the application of the DTBM-SEGPHOS-based copper-hydride methodology to the regio- and stereoselective synthesis of chiral secondary amines (Scheme 28) [79]. The introduction of a more electron rich ester increases the stability of the amination reagent over its decomposition by the copper-hydride complex and consequently affords higher yields of secondary amines. A variety of chiral secondary amines was accessible by this modified protocol from a range of functionalised and structurally diverse amine transfer reagent and mono- and disubstituted styrenes.

**Scheme 29:**
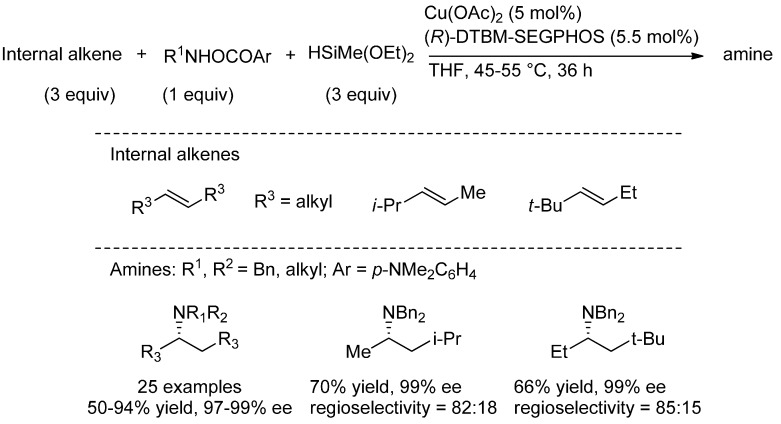
Regio- and enantioselective copper (I) hydride catalysed electrophilic amination internal alkenes.

The same year, this copper-based formal hydroamination process was extended to the regio- and enantioselective transformation of internal olefins as extremely challenging unactivated alkenes [80]. It was observed that the employment of amine transfer reagents bearing 4-(dimethylamino) benzoate group were also crucial to productively obtain chiral amines in high yields. The use of these electronically enriched esters in conjunction with once more the catalytic system Cu(OAc)_2_·(*R*)-DTBM-SEGPHOS and in presence of diethoxymethylsilane delivers chiral amines in impressively high level of enantioinduction from a variety of symmetrical internal olefins as displayed in Scheme 29. In the case of unsymmetrical internal alkenes, a modest level of regioselectivity was noticed despite the high optical purity of each regioisomer. The major isomer may result from electrophilic amination at the more sterically available alkylcopper intermediate. It was shown by DFT calculations that the spatial arrangement of the aryl groups of the bulky diphosphine ligand controls the facial approach of the alkene during the key enantio-determining hydrometalation step.

**Scheme 30:**
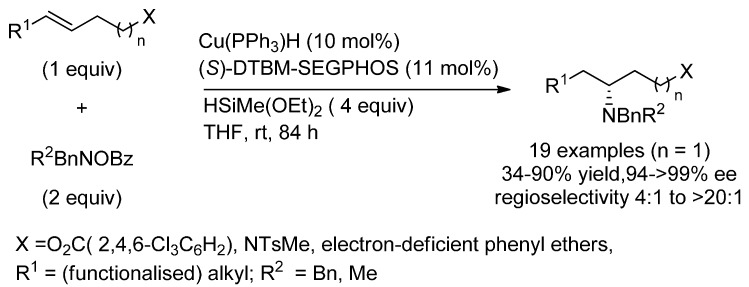
Enantioselective and electronically controlled regioselective copper (I) hydride catalysed electrophilic amination of homoallylic esters.

Last year, Hartwig and co-workers brilliantly broadened the DTBM-SEGPHOS-based copper–hydride methodology to the highly regio- and enantiocontrolled formal hydroamination of internal olefins functionalised at the homoallylic position by an oxygen or nitrogen atom substituted by an electron-withdrawing group (Scheme 30) [81]. Screening investigations on the reactivity of oxygen-containing internal olefins and *O*-benzoyl-*N*,*N*-dibenzylhydroxylamine emphasised that the reaction regioselectivity and the hydroamination yield were both influenced by the steric and electronic factors of the group attached to the oxygen atom placed at the homoallylic position. The electron-deficient 2,4,6-trichlorobenzoyl group on the oxygen atom provided better results in terms of yield and regioselectivity. A variety of functionalised (*E*)-1,2-dialkylolefins bearing the 2,4,6-trichlorobenzoyl group or electron-deficient phenyl ethers were converted to the corresponding chiral amines by this procedure based on the DTBM-SEGPHOS–Stryker reagent combination as catalyst (Scheme 30). However, the olefin geometry and position from the oxygen or nitrogen atom as well as the nature of the electrophilic amination reagent strongly impact the outcome of the transformation. Indeed, the reaction of (*Z*)-1,2-dialkylalkenes or alkenes bearing a longer spacer chain (*n* = 2, Scheme 30) provide low yields of formal hydroamination products. It is worth noting that the high regiocontrol is dictated by the electronic effect of the 2,4,6-trichlorobenzoyl substituent on the C-C double bond rather than direct coordination of the ester function to the copper catalyst.

Overall, the highly catalyst-controlled diastereoselectivity and the broad functional group tolerance of the DTBM-SEGPHOS-based copper-hydride methodology offer wide potential applications in the late-stage syntheses of bioactive pharmacologically relevant compounds [79,81]. Additionally, the high reliability, predictability and control of this CuH-methodology open the door to its incorporation into different auto-tandem catalytic processes [82] as elegantly illustrated by the Buchwald group [83,84,85]. Indeed, the integration of this hydroamination methodology into tandem processes provides access to various classes of (chiral) amines (linear alkylamines, chiral 1,3-amino alcohols, enamines or α-, γ-, or δ-chiral branched alkylamines), from easily accessible compounds (allylic esters, allylic alcohols, allylic ethers, enones, enals and alkynes) with high levels of chemo-, regio-, and stereoselectivity. However, despite these tremendous advances, the overall atom and step efficiency of this formal hydroamination strategy digresses remarkably from the original concept of the hydroamination reaction. Indeed, this strategy demands the prior preparation of complexed electrophilic amination reagents additionally to the use of a large excess of silane reducing agent. On this point, this CuH-methodology is more relevant to transition-metal-catalysed hydrosilylation reactions [86] than classical hydroamination reactions.

### 6.2. Hydroalkoxylation

**Scheme 31:**
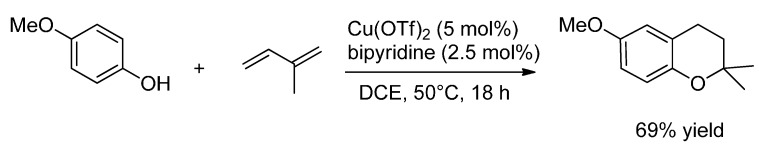
Addition of 4-methoxyphenol to isoprene catalysed by a Cu(OTf)_2_-bipyridine system.

In 2005, Hii et al., evidenced the usefulness of a copper catalytic system in the regioselective addition of ROH to norbornene [87]. The group has also studied the association of copper(II) triflate-bipyridine in the annulation of phenols and naphthol with 1,3-dienes. This air- and moisture-stable catalyst allows a tandem hydroalkoxylation-rearrangement-hydroalkoxylation sequence and produces benzopyran derivatives in aerobic conditions as exemplified for isoproprene in Scheme 31 [88]. The authors mentioned that this catalyst can be recycled and reused at least three other times without loss of activity. The initiation step of the hydroalkoxylation reaction seems to be initiated by the formation of TfOH.

By comparing copper triflate with other metal triflates on the addition of 2-hydroxyethyl methacrylate to dicyclopentadiene, the Carpentier group showed in 2009 that both copper and triflate ions are necessary for efficient catalytic activity [89]. In presence of a hydrogen donor, copper(II) triflate is oxidised in copper(I) triflate involving that the liberated triflic acid promotes efficiently the reaction. Moreover, both CuOTf and Cu(OTf)_2_ play an important role of inhibition of the polymerisation of the reaction product.

**Scheme 32:**
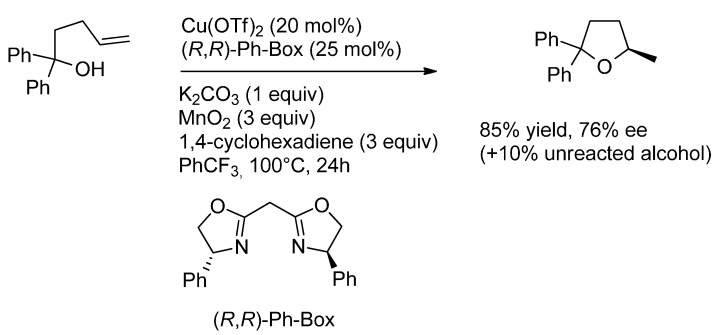
Enantioselective intramolecular hydroalkylation of alkenes catalysed by a chiral copper complex.

In 2012, during a study on the intramolecular carboetherification of alkenes, Chemler et al., described a formal intramolecular hydroalkoxylation proceeding via an oxycupration step [19]. The radical intermediate is then protonated in presence of 1,4-cyclohexadiene. Copper(II) triflate associated to (*R*,*R*)-Ph-Box leads to the corresponding tetrahydrofuran with 76% ee; even if it is applied to only one substrate, this result is the first example of asymmetric Markovnikov hydroalkoxylation of an unactivated alkene (Scheme 32). It is important to note that the enantioselective transannular hydroetherification vs. carboetherification depends on the presence or not of 1,4-cyclohexadiene in the reactional medium.

**Scheme 33:**
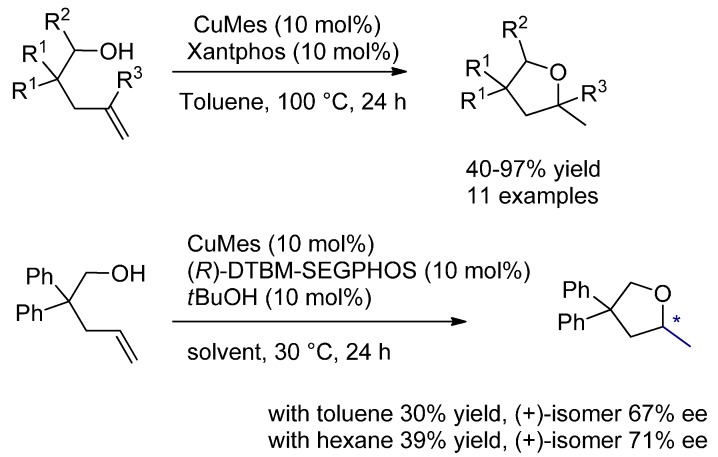
Intramolecular hydroalkoxylation reaction catalysed by mesityl copper (I) in presence of Xantphos or (*R*)-DTBM-SEGPHOS.

In 2015, Sawamura et al., studied the intramolecular hydroalkoxylation of unactivated terminal alkenes catalysed by a complex of Cu (I)-Xantphos (Xantphos, Figure 2) (Scheme 33) [90]. This new copper catalyst promotes the reaction according to a mechanistically different pathway from other copper catalysts previously reported. The mesitylcopper(I) activates the substrate to form an alkoxocopper(I) intermediate bearing a Cu-O bond. Subsequent alkene insertion into the Cu-O bond provides an alkyl copper complex. Then, the resulting complex undergoes alcoholysis from the substrate to give the desired cyclic ether releasing the alkoxocopper(I) species. A ligand screening was carried out with 2,2-diphenyl-4-penten-1-ol as unique substrate and revealed that Xantphos (Figure 2), having a large bite angle, is the most effective. This Cu-Xantphos complex is able to react not only with the primary alcohols but also with the secondary ones. A chiral diphosphine ligand screening indicates that (*R*)-DTBM-SEGPHOS is as enantioselective as (*R*)-DTBM-BINAP or (*R*)-DTBM-BIPHEP. However the copper-(*R*)-DTBM-SEGPHOS is the more active since at 60 °C it leads to the desired cyclic ether with 94% yield and 51% ee. In order to improve the enantioselectivity, the reaction temperature is decreased to 30 °C; the enantiomeric excess is then increased to 67% with a dramatic lowering of the yield (30%) (Scheme 33). From this result, the enantioselectivity can be increased whether toluene is changed by hexane leading to the chiral tetrahydrofuran derivative with 71% ee. It is worth to note that as for the previous copper catalytic system, the application of the asymmetric version of the complex was reported only for a single substrate.

**Scheme 34:**
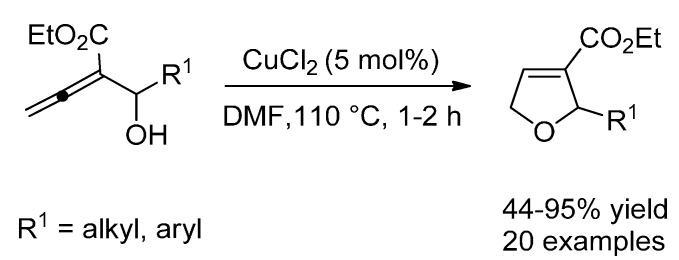
Copper-catalysed intramolecular hydroalkoxylation of allenes by a selective 5-*endo* cyclisation.

Intramolecular hydroalkoxylation of α-hydroxymethylallenes is one way to prepare 2,5-dihydrofurans. In 2012, Lee et al., developed a method lying on the use of copper(I) and copper(II) halides as catalysts in the cyclisation of ethyl α-(1-hydroxy-1-alkyl)methylallenoates and α-(1-hydroxy-1-aryl)methylallenoates [91]. The best conditions are found with CuCl_2_ (5 mol %) in DMF at 110 °C (Scheme 34). The cyclisation proceeds through a 5-*endo* mode whereas a 6-*endo* hydroarylation is generally observed with Au (I) species. Intramolecular copper-catalysed hydroalkoxylation would be initiated by the activation of the allenyl group by the copper species followed by the attack of the hydroxy group according to a 5-*endo* cyclisation. The vinylcopper intermediate after liberation of HX would lead to the 2,5-dihydrofuran derivative and would generate copper to proceed with the catalytic process.

### 6.3. Hydrophosphination

**Scheme 35:**
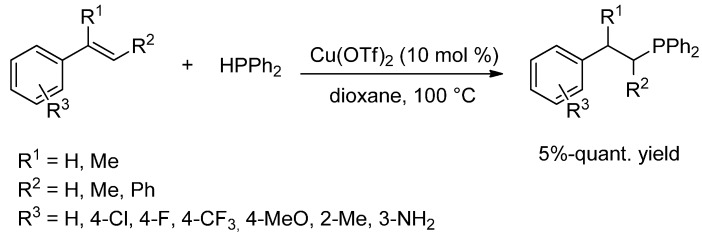
Copper-catalysed intermolecular hydrophosphination of styrene derivatives.

As reported by the Corma group [24], copper(II) triflate was efficient as catalyst in alkene hydrophosphination reactions (Scheme 35). Exclusive *anti*-Markovnikov adducts are obtained starting from variously substituted styrenes. Yields are ranging from poor to quantitative within these series. In contrast, non-activated alkenes such as stilbene or 1-octene afforded low yields of hydrophosphination product.

## 7. Zinc

### 7.1. Hydroamination

**Scheme 36:**
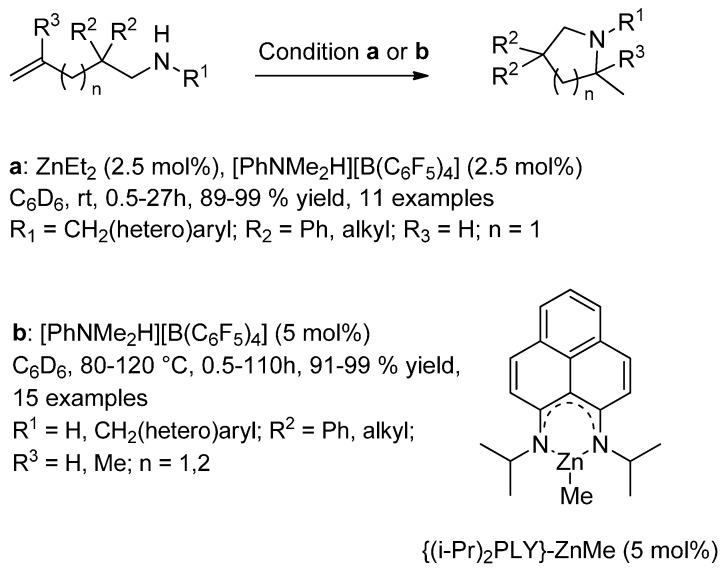
Zinc-catalysed intramolecular alkene hydroamination of primary amines.

In 2005, the first application of a zinc-derived catalyst for alkene hydroamination was reported by the Roesky group using [*N*-isopropyl-2-(isopropylamino)troponiminato] methylzinc complex as catalyst in the presence of [PhNMe_2_H][B(C_6_F_5_)_4_] as co-catalyst [92]. A year later, replacement of the isopropyl groups of the complex by cyclohexyl groups delivered a more stable and active version of the zinc alkyl catalyst [93]. The latter affords good catalytic activities at high temperature for the preparation of a variety of functionalised five-membered nitrogen heterocycles by alkene hydroamination. Further modulations of the steric and electronic properties of the aminotroponimate skeleton have highlighted a pronounced effect of these parameters on the stability and activity of these complexes [94,95,96]. Unfortunately, these in depth investigations have not identified a more reactive complex than the one bearing the cyclohexyl groups on the nitrogen atom of the ligand. In 2009, the same research group disclosed a very useful ligand-free protocol for the zinc-catalysed room temperature hydroamination of secondary amines tethered to terminal alkenes as described in Scheme 36 (Condition **a**). In this protocol, the combination of commercial ZnEt_2_ (2.5 mol %) and [PhNMe_2_H][B(C_6_F_5_)_4_] (2.5 mol %) as activator allows the formation of pyrrolidines at room temperature from aminoalkenes biased towards cyclisation and at higher temperature for those free of Thorpe-Ingold effect. This simple catalytic system exhibits the highest activity so far for a zinc-based system. It is important to note that the coordination ability of the activator anion strongly modifies the system reactivity. More recently, a well-defined alkyl zinc complex supported by a symmetrical *N*-isopropyl-substituted phenalenyl-based ligand was reported by Mandal and co-workers [97,98]. This complex, in the presence of [PhNMe_2_H][B(C_6_F_5_)_4_], displays similar catalytic activity to that of Roesky’s [*N*-cyclohexyl-2-(cyclohexylamino)troponiminato] methylzinc complex for the conversion of functionalised primary and secondary aminoalkenes into five- and six-membered rings (Scheme 36, Condition **b**).

In 2009 the first and only report of enantioselective zinc-promoted alkene hydroamination using a bis(organozinc) complex supported by a chiral l-proline modified diamidobinaphthyl ligand was published by the Hultzsch group [99]. Beside the fact that the observed enantiomeric excesses for the cyclised products were moderate (<29% ee), the proof-of-principle was clearly established and deserves to be highlighted.

### 7.2. Hydroalkoxylation

**Scheme 37:**
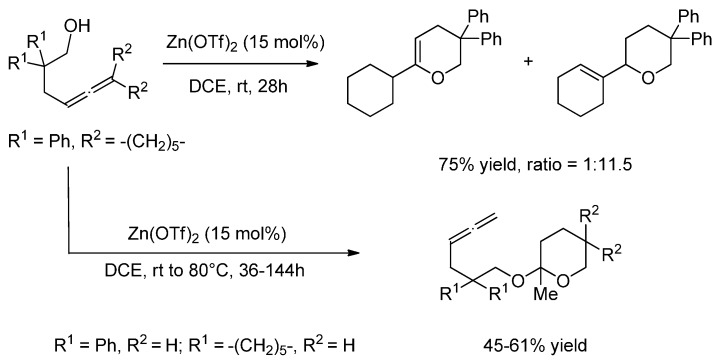
Zn(OTf)_2_-catalysed 6-*exo*-dig cyclisation of γ-allenols.

To the best of our knowledge, zinc has only been reported as a metal catalyst for intramolecular hydroalkoxylation of allenols by Hii et al. in 2009 (Scheme 37) [100]. In the intramolecular hydroalkylation of γ-allenols which can afford 5- or 6-membered *O*-heterocycles, a regiodivergence was observed depending on the metal catalyst used. When the reaction is catalysed by Zn(OTf)_2_ (or Sn(OTf)_2_) 6-*exo*-dig cyclisation occurs mostly whereas catalysis proceeds by 5-*exo*-trig cyclisation in the presence of AgOTf. The authors noted that 6-*exo*-trig cyclisation was previously reported only with lanthanides, whereas the more common 5-*exo*-trig selectivity was also observed with gold and platinum catalysts. Depending on the substrate structure, (for example when R^1^ = Ph or -(CH_2_)_5_- and R^2^ = H), the hydroalkoxylation step resulting in the formation of a 6-membered ring cyclic ether intermediate which could be trapped by another molecule of substrate to give an acetal.

## 8. Conclusions

This review has highlighted some of the most recent advancements in the addition of E-H bond (E = N, O, P) onto unactivated alkenes catalysed by first-row late transition metal-based complexes derived from manganese, iron, cobalt, nickel, copper and zinc. By far, most of the research activity has been focused on the development of catalytic systems for the C-N bond formation certainly due the predominance of nitrogen-containing scaffolds in biologically active molecules. Conversely, less attention has been paid to the formation of C-O bond and to a lesser extent to the formation of C-P bond by this methodology. Although the use of these earth-abundant elements in alkene hydrofunctionalisation is still in its infancy, the current scientific interest for these elements has already led to some outstanding achievements and landmarks in this burgeoning field. For example, the emergence of the notorious diphosphine copper-hydride chemistry and metal-mediated radical processes into the area of alkene hydroamination has already successfully unraveled some remaining limitations in terms of applicability, reaction scope, chemo-, regio- and enantioselectivity and catalyst efficiency. However, these formal approaches are in a way antagonist to the original concept of hydrofunctionalisation reaction from the viewpoint of atom preservation and progress are still needed towards atom efficiency. Some advances in this direction have been proposed through the use of a more classical “truly” hydroamination strategy and gain to be noticed and pursued. Another striking feature that arises from these studies is the challenging difficulty to control the regioselectivity of the addition to independently access Markovnikov and *anti*-Markovnikov adducts that are both compounds of interest. As a general trend, the addition reactions of N-H and O-H onto unactivated alkenes developed so far afford preferentially the Markovnikov adduct, but some advances to access the other regioisomer have been reported and are worth noting. In contrast, the C-P addition reactions usually proceed with *anti*-Markovnikov selectivity although that ligand-free iron chloride salts have proven the ability to provide both adducts exclusively, depending on the oxidation state of the metal. As far as we know, sporadic examples of enantioselective alkene hydroalkoxylation and no example of alkene hydrophosphination, hydro-phosphonylation or hydrophosphinylation have been reported yet. The latter point may be related to the preferred *anti*-Markovnikov regioselectivity generally demonstrated for the C-P bond formation. The development of catalytic, enantiocontrolled additions of E-H (E = O, P) on unactivated alkenes promoted by first-row late transition metal is highly desirable but still remains very challenging. It is manifest that first-row late transition metals show great potential and prospects for the C-N, C-O and C-P bond construction by E-H (E = N, O, P) addition reactions onto unactivated alkenes. Their one- and two-electron reactivity will undoubtedly be key in enabling novel reactivity and selectivity in the area of hydrofunctionalisation reactions. In the future, the attractive challenge lies in the control of this dual reactivity by an accurate ligand design.
